# Revisiting Organisational Learning in Integrated Care

**DOI:** 10.5334/ijic.3047

**Published:** 2017-08-11

**Authors:** Roberto Nuño-Solinís

**Affiliations:** 1 Deusto Business School Health, University of Deusto, Bilbao, ES

**Keywords:** Integrated care, organisational learning, healthcare integration, knowledge management, organisational change

## Abstract

Progress in health care integration is largely linked to changes in processes and ways of doing. These changes have knowledge management and learning implications. For this reason, the use of the concept of organisational learning is explored in the field of integrated care.

There are very limited contributions that have connected the fields of organisational learning and care integration in a systematic way, both at the theoretical and empirical level. For this reason, hybridization of both perspectives still provides opportunities for understanding care integration initiatives from a research perspective as well as potential applications in health care management and planning.

## Introduction

Organisational learning is a concept developed over the last four decades in the business management literature, although its track record in the health field is not as extensive as in other sectors of activity. This acknowledgement of the limited contribution of organisational learning to healthcare in general and to integrated care, in particular, justifies it being chosen as a “lost” topic in the literature which could be of interest to the readers of the *International Journal of Integrated Care*. We, therefore, aim to revisit the concept of organisational learning and study its progress over the last few decades.

As authors like Edmonson and Moingeon [1] recognised organisational learning encompasses a too broad territory in the management literature that is prone to confusion and multiple meanings. Therefore, they defined organisational learning as a process in which an organisation’s members actively use data to guide behaviour in such a way as to promote the ongoing adaptation of the organisation. In this paper, we use this definition that allows understanding organisational learning as a process that can be initiated, developed, and practised.

The first papers in this field appeared in the 1960s with pioneering contributions from March and Simon [2], and Cyert and March’s general theory of organisational learning and organisational routines [3], but the topic became popular as a result of the theory of action by Argyris and Schon [4] in the late 1970s, culminating in a veritable explosion of management publications on organisational learning in the 1990s with a parallel boom in the number of authors interested in the subject, including the contributions by Peter Senge [5], which represent a genuine turning point in this field. In his book “The Fifth Discipline: The Art and Practice of the Learning Organisation”, he considered organisational learning to be a key element in the overall understanding of the organisation and the relationships between its components, defining it from a systemic perspective and highlighting the importance of leadership in this field. He also put forward the concept of the Learning Organisation, which subsequently acquired great popularity.

The following years saw the consolidation of the body of knowledge concerning learning within organisations that is relevant for health organisations [6] and for integrated care.

As integrated care does not evolve as a natural response to emerging care needs in any system of care, whether planned or market-driven, great relevance is also acquired by the study of organisational learning as a deliberate, proactive process [7,8] more than a mere process of adaptation [3] to the requirements of the surrounding environment. Successful organisations are therefore those that are capable of anticipating changes in the environment, learning proactively and redesigning their processes with a view to obtaining the desired results. This capacity to learn and improve performance is framed within a knowledge-intensive sector with a pace of technology innovation and production of new biomedical knowledge scarcely paralleled in other fields of activity. Moreover, it is confronted with demographic and epidemiological challenges characterised by chronicity and multimorbidity.

The current context of integrated care development is ideal for revisiting the contributions that theories such as organisational learning can make in this regard. In the US, for example, in the field of development of ACOs (Accountable Care Organisations), Shortell [9] and Nembhard and Tucker [10] acknowledged that organisational learning can improve the understanding of how organisations collaborate, learn from each other, and achieve coordination. It also aids understanding of the dynamics of piloting and incorporation of new behaviours and practices geared to integration and their impact on the progress towards the Triple Aim [11].

On researching the literature for study in-depth the organisational learning concept applications related to integrated care, we discovered an early work that had not been widely disseminated, published under the title of “The Integrated Care in Shropshire project: lessons from experience” [12]. This is one of the pioneering contributions that highlighted the role of organisational learning in care integration projects and in relation to interprofessional and interorganisational collaboration, teamwork, development of a shared vision, etc. As the paper was written in the early 1990s (coinciding with the popularisation of the concepts of organisational learning and the Learning Organisation in the management literature) the mention of this concept comes as no surprise; what it is surprising is that it subsequently reappears so little in the field of integrated care. In fact, it was not until 1998 [13] that a paper was published contributing to theoretical advancement in both areas of knowledge, suggesting how integrated delivery systems can create a climate of system-wide learning through a shared vision, facilitative leadership and an organic structure.

It will also be seen that some of the core issues of integrated care development and implementation have been described in the literature for several decades but have not been generally resolved [14], despite the large amount of experiences, knowledge and evidence of effectiveness built up on the subject [15].

## Description of the organisational learning concept

The working definition of organisational learning used in this paper considers it as a process that requires individual cognition and supports organisational adaptiveness. It is a process of acting, assessing, and acting again, an ongoing cycle of reflection and action that cannot be taken for granted in organisations [1].

Organisations thus improve their processes and products as they learn, integrating new knowledge, and this enables them to perform successfully in a changing environment [16,17]. It has also been observed that companies’ per-unit production costs tend to decrease over time [18]. This cost reduction is attributed to learning. The relationship between time elapsed and improvement in performance has been called “the learning curve”.

In competitive markets, much of the research has focused on organisations that learn from their own experience in order to gain a competitive advantage and on studying why some organisations in rapidly-changing industries learn, adapt and prosper while others do not [19,20,21]. Keeping up with the pace of technology innovation strains the organisations to such an extent that trusting in experiential adaptation (based on their accumulated experience) does not guarantee their competitiveness or survival. Therefore the aforementioned notion of “deliberate” organisational learning emerges, meaning that re-engineering is required to produce the best results.

The organisational learning theory takes the socio-organisational context into account, and the individuals in an organisation thus learn within a social context where others are also learning, immersed in both previously acquired knowledge and accumulated learnings. From a theoretical perspective, Argyris and Schon [4] distinguish three types of organisational learning that are essential to understand and analyse in order to overcome the fragmentation of care. First, simple or single-loop learning refers to corrective actions implemented in response to shortcomings in the organisation’s activity. Second, double-loop learning connects and links “knowledge with understanding and reasoning for action”, and triple-loop learning involves learning about the actual learning processes. Organisations committed to triple-loop learning will have few limitations for understanding the relationship between action and results, and they will be ahead of the rest as regards adaptation capacity and unlearning the old ways of doing things, these being key aspects for developing care integration.

## Discussion of its impact

As it has already been mentioned, the concept of organisational learning does not have such an extensive track record in the health field as in other sectors, although organisations such as the Institute of Medicine [22,23,24] have highlighted its relevance in the health area.

If we focus on the area of knowledge of care integration, the impact of organisational learning theories is even more limited. Although there have been some relevant contributions, most are exploratory and conceptual approaches, and except for a paper by van Wijngaarden et al [25], we have found no other empirical studies.

In fact, van Wijngaarden’s group conducted a qualitative study from an organisational learning perspective within an integrated network of care provision for stroke patients in the Netherlands. It mainly centred on the professionals’ management of organisational boundaries in the patients’ transitions. Their findings show the importance of relational and collaborative elements with regard to joint learning between professionals from different organisations. They also show that knowledge coding tools such as routes and protocols are useful, but that they need to be complemented by others enabling tacit knowledge transfer. This paper contains a valuable and relevant practical application of the organisational learning perspective in care integration.

On a theoretical level, Tsasis et al [26] explicitly proposed conceptualising integration as a learning process and the organisations and professionals as players who learn within a framework of complex adaptive systems. They particularly stress the need to understand the phenomenon of learning and its barriers and facilitators in the context of cross-disciplinary groups of professionals. Finally, they highlight the capacity of learning to learn as a key element in the organisations’ effective performance. They thus bring together four lines of knowledge: organisational learning, integrated care, the complexity theory and the theory of “learning organisations”. It is a theoretical contribution of great importance, but it has not been developed empirically.

Other authors have analysed care integration from the perspective of collaboration and coordination within and between healthcare providers and the role of learning in healthcare. Internal collaboration is complex in itself, as transparency is required and defensive routines must be overcome [20], but external collaboration is an even greater challenge, particularly when the entities have different approaches and ways of working (primary healthcare vs. hospitals, for example), different goals and they may even have been former competitors [27].

Despite this scarcity of contributions, the enormous potential of organisational learning for the development of integrated care should be highlighted, for example:

*From a knowledge management perspective*. As Davenport and Prusak [28] stated: “Knowledge management is managing the corporation’s knowledge through a systematically and organisationally specified process for acquiring, organising, sustaining, applying, sharing and renewing both the tacit and explicit knowledge of employees to enhance organisational performance and create value”. Therefore organisational learning theory is a fundamental metatheory for understanding the knowledge transfer phenomena inherent to the development of integrated care. The members of an organisation interact to add meaning and knowledge to the relationship between a determined action and the result obtained, and also to the effects of the context of their organisation (i.e. learning environments) on this relationship. In this regard, it is particularly important to be able to analyse whether the organisational and cultural profiles are relevant for better learning, i.e. whether there are differences between intra- and inter-organisational learning and which elements act as barriers and facilitators in each case, such as integration initiatives, with regard to either processes or structures. The nature of the knowledge is also relevant, i.e. whether it is tacit or coded, as developing care integration requires advancement in both types of knowledge [25] like guidelines, protocols, shared medical records, etc. on the one hand, and routines, values, ways of doing, etc. on the other.

*From the perspective of organisational identity*. The development of vertical integration, either virtual, through ACO agreements or similar, or real, with the creation of single-ownership IHOs (Integrated Healthcare Organisations), enables a comparative analysis to be made of the intra- and inter-organisational learning processes and whether these have any differentiating features in organisations with a unique or different “identity”. The traditional experts’ consensus can be summed up in the well-known statement by Burns and Pauly [29] “integrated care structures rarely integrated the actual delivery of patient care”, and the subsequent literature on the subject [30] is fairly consistent with regard to showing that mere vertical integration via merger or absorption does not guarantee greater clinical integration or better health outcomes [31] or better economic outcomes [32], although it is not totally conclusive [33]. This issue, therefore, merits more detailed analysis, not only from the organisation’s legal perspective but also in terms of its identity and the relationship between identity and learning. Organisational identity is referred to as the character of an organisation that is considered central, distinctive and enduring and it is also deeply linked to organisational culture because it is grounded in organisational symbols and local meanings. Several authors have demonstrated that groups of people with strong organisational identification have greater intentions to stay with a firm, perform better, and are more cooperative [34,35].

*From the perspective of process redefinition and optimisation*. The change from fragmented processes to patient-centered integrated processes is one of the essential transformations in achieving integrated care, in the words of Shortell et al [36] “to offer a coordinated continuum of services to a defined population”. With very few exceptions, it seems that in the health sector more progress has been made in single-loop learning (the incremental improvement of existing processes at the operational level) than in double-loop learning (process redesign) or deutero-learning (learning to learn). For example, in many quality improvement initiatives, the single-loop learning approach dominates assuming that problems and their solutions to be close to each other in time and space. Transformation towards integrated care requires a shift to a systemic perspective questioning the underlying way that “things” has been done and thus calls for double-loop learning.

*From the perspective of organisational ambidexterity*. The term ambidexterity is used to refer to the need to achieve a balance between different forms of learning. An ambidextrous organisation is thus an organisation able to combine different forms of learning. An article by March, published in 1991, states that exploration and exploitation involve two different forms of organisational learning [37]. Exploitation involves learning geared to detection and correction of errors (enabling improvement of the existing organisational processes), as opposed to learning geared to questioning the theories-in-use or basic starting assumptions, enabling these processes to be renewed. The organisational processes are not questioned in the former case, unlike in the latter case where this questioning may give rise to their total renewal. In the case of integrated care, the incremental improvement of processes built in silos (primary care vs. hospitals; public health vs. healthcare, healthcare vs. social care, etc.) contributes to the efficiency of organisational units but not to that of the actual system of care. Therefore, the development of exploratory capacities is required. This entails behaviour geared to flexibility, searching, change, variation, risk, experimentation, play, discovery or innovation. Exploration is characterised by the reorientation [38] of routines and organisation processes and by the search for new rules, technologies, goals and purposes, rather than merely learning to develop the existing routines more efficiently. Similarly, as knowledge regeneration occurs, exploration also includes abandoning obsolete or useless knowledge, i.e. “unlearning” [39]. The perdurance of a wide translational gap between research and practice in the health sector allows us to conjecture that the provider organisations have made more progress in exploiting knowledge than in exploring it. Integrated care development may therefore be linked to the development of exploratory capacities within the provider organisations, working on organisational ambidexterity. Many of the successful integration experiences have been conducted as part of pilots or one-off projects, often in contexts permitting innovation outside the existing “rules” and organisational inertias. However, the scalability of pilot projects to other organisations or larger organisational units continues to pose challenges which the organisational learning literature can help to understand and resolve.

*From the perspective of learning organisations*. It is noteworthy that most organisations identified in the literature as health sector learning organisations are integrated health systems such as Kaiser Permanente, Geisinger, VHA or Intermountain [24,40,41]. A distinguishing feature of these high-performing organisations in environments of great uncertainty such as the health sector is their capacity to involve their professionals in higher-order learning processes. Organisations committed to triple-loop learning processes will have few limitations for understanding the relationship between action and results, and they will be advantaged with regard to adaptation capacity, resilience and sustainability.

## Key lessons

Transformation towards integrated care can be conceptualised as an organisational learning experience. This transformation requires collaborative learning between cross-disciplinary teams and different care levels, and often between different healthcare organisations. It is essential to know and understand these dynamics and observe how they are influenced by professional cultures and the identities and cultures of the organisations.

Organisational learning within a context of care integration is linked to the achievement of better results, but the learning curve can show reversals at the initial stage, as it is necessary at this stage to invest in exploration activities in detriment to exploitation activities [10]. Achieving a trajectory of positive results requires successful implementation of proven innovations and an extensive time frame. This is the case for many of the success stories cited in the literature [42].

Finally, cross-fertilisation between organisational learning, theories of innovation and quality improvement, complexity science and implementation research may facilitate major progress in understanding care integration and developing interventions for its advancement (best practice sharing, design of learning environments, interorganisational collaboratives, communities of practice, different types of training activities, internal problem solving, leadership development, etc.) and instruments for measuring them, like the Learning Organization Survey developed by Singer et al that has been applied in healthcare with valuable results [43].

## Conclusion

This paper has explored the historical evolution of how the concept of organisational learning has been used in the field of care integration and has drawn some useful conclusions for practical implementation of the concept and research in this area of knowledge.

This study has the limitations of the sources of information consulted and the non-unequivocal nature of the guiding concepts, particularly those related to care integration, for which a large number of similar or related concepts exist [44].

We have confirmed that the application of the organisational learning theory in order to understand care integration processes, strategies, interventions and initiatives has been very limited up until now, despite the pioneering contributions that have been made since the 1990s, most of which remain valid today. This paper’s main contribution is the recovery of these contributions, combining them with some more recent ones that advocate the role of organisational learning in the understanding of care integration processes.

To sum up, the concept, theories and methods of organisational learning have great potentialities from the perspectives of both research and management, and they may help us to understand what has become known as the “black box” [45] of care integration initiatives. In the present context, where initiatives for developing integrated care proliferate in many health systems around the world, its future use is a promising prospect. We trust that we will not have to wait another two decades to obtain useful results.

## Declaration of transparency

The lead author (the manuscript’s guarantor) affirms that this manuscript is an honest, accurate and transparent account of the study sent to IJIC, that no important aspects of the study have been omitted and that any discrepancies from the study as planned (and, if relevant, registered) have been explained.

## References

[1] Edmondson A, Moingeon B (1998). From organizational learning to the learning organization. Manag Learn.

[2] March J, Simon HA (1958). Organisations.

[3] Cyert R, March JG (1963). A Behavioral Theory of the Firm.

[4] Argyris C, Schön D (1978). Organisational learning.

[5] Senge PM (1990). The Fifth Discipline: The Art and Practice of the Learning Organisation.

[6] Bohmer RM, Edmondson AC (2001). Organisational learning in health care. Health Forum J.

[7] Dutton JM, Thomas A (1984). Treating progress functions as a managerial opportunity. Academy of Management Review.

[8] Nembhard IM, Cherian P, Bradley EH (2014). Deliberate learning in health care: The effect of importing best practices and creative problem solving on hospital performance improvement. Medical Care Research and Review.

[9] Shortell SM (2016). Applying organisation theory to understanding the adoption and implementation of accountable care organisations. Medical Care Research and Review.

[10] Nembhard I, Tucker AL (2016). Applying organisational learning research to accountable care organisations. Medical Care Research and Review.

[11] Berwick DM, Nolan T, Whittington J (2008). The triple aim: Care, health, and cost. Health Affairs.

[12] Waddington P (1994). The Integrated Care in Shropshire project: lessons from experience. Br J Nurs.

[13] Barnsley J, Lemieux-Charles L, McKinney MM (1998). Integrating learning into integrated delivery systems. Health Care Manage Rev.

[14] Goodwin N (2016). Understanding and Evaluating the Implementation of Integrated Care: A ‘Three Pipe’ Problem. International Journal of Integrated Care.

[15] Martínez-González NA, Berchtold P, Ullman K, Busato A, Egger M (2014). Integrated care programmes for adults with chronic conditions: a meta-review. International Journal for Quality in Health Care.

[16] Crossan MM, Lane HW, White RE (1999). An organisational learning framework: from intuition to institution. Acad Manage Rev.

[17] Garvin DA (2000). Learning in action: A guide to putting the learning organisation to work.

[18] Abernathy WJ, Wayne K (1974). Limits of the learning curve. Harvard Business Review.

[19] Argote L (2012). Organisational learning: Creating, retaining and transferring knowledge.

[20] Argyris C (1990). Overcoming organisational defenses: Facilitating organisational learning.

[21] Huber GP (1991). Organisational learning: The contributing processes and the literatures. Organisation Science.

[22] Institute of Medicine (2004). Keeping patients safe: Transforming the work environment of nurses.

[23] Institute of Medicine (2011). Engineering a learning healthcare system: A look at the future: Workshop summary.

[24] Institute of Medicine (2012). Best Care at Lower Cost: The Path to Continuously Learning Health Care in America.

[25] van Wijngaarden J, de Bont A, Huijsman R (2006). Learning to cross boundaries: The integration of a health network to deliver seamless care. Health Policy.

[26] Tsasis P, Evans JM, Rush L, Diamond J (2013). Learning to learn: towards a relational and transformational model of learning for improved integrated care delivery. Adm Sci.

[27] Conrad DA, Grembowski D, Hernandez S, Lau B, Marcus-Smith M (2014). Emerging lessons from regional and state innovation in value-based payment reform: Balancing collaboration and disruptive innovation. Milbank Quarterly.

[28] Davenport TH, Prusak L (2000). Working Knowledge: How Organizations Manage What they Know.

[29] Burns LR, Pauly MV (2002). Integrated delivery networks: a detour on the road to integrated health care?. Health Aff (Millwood).

[30] Evans J, Baker R, Berta W, Barnsley J, Goes J, Savage G, Friedman L (2013). The evolution of integrated healthcare strategies. Annual review of health care management: revisiting the evolution of health systems organisation advances in health care management.

[31] Carlin CS, Dowd B, Feldman R (2015). Changes in quality of health care delivery after vertical integration. Health Services Research.

[32] Goldsmith J, Burns LR, Sen A, Goldsmith T (2015). Integrated delivery networks: In search of benefits and market effects. National Academy of Social Insurance.

[33] Hwang W, Chang L, LaClair M, Paz H (2013). Effects of integrated delivery system on cost and quality. American Journal of Managed Care.

[34] Ellemers N, de Gilder D, van den Heuvel H (1998). Career-oriented Versus TeamOriented Commitment and Behavior at Work. Journal of Applied Psychology.

[35] Jetten J, O’Brien A, Trindall N (2002). Changing Identity: Predicting Adjustment to Organisational Restructure as a Function of Subgroup and Superordinate Identification. British Journal of Social Psychology.

[36] Shortell SM, Gillies RR, Devers KJ (1995). Reinventing the American hospital. Milbank Quarterly.

[37] March JG (1991). Exploration and exploitation in organisational learning. Organisation Science.

[38] Lant T, Mezias S (1992). An Organisational Learning Model of Convergence and Reorientation. Organisation Science.

[39] Hedberg B, Nystrom C, Starbuck W (1981). How organisations learn and unlearn. Handbook of organisational design.

[40] Psek WA, Stametz RA, Bailey-Davis LD, Davis D, Darer J, Faucett WA (2015). Operationalizing the learning health care system in an integrated delivery system. EGEMS (Wash DC).

[41] Nuño-Solinís R (2015). Kaiser Permanente: ¿qué se puede aprender de su experiencia en integración asistencial?. Revista de Innovación Sanitaria y Atención Integrada.

[42] Kizer KW, Dudley RA (2009). Extreme makeover: Transformation of the Veterans health care system. Annu Rev Public Health.

[43] Singer SJ, Moore SC, Meterko M, Williams S (2012). Development of a short-form Learning Organisation Survey: the LOS-27. Medical Care Research and Review.

[44] Valentijn PP, Boesveld IC, van der Klauw DM, Ruwaard D, Struijs JN, Molema JJ (2015). Towards a taxonomy for integrated care: a mixed-methods study. International Journal of Integrated Care.

[45] de Stampa M, Vedel I, Bergman H, Novella JL, Lechowski L, Ankri J, Lapointe L (2013). Opening the black box of clinical collaboration in integrated care models for frail, elderly patients. Gerontologist.

